# Energy drinks at adolescence: Awareness or unawareness?

**DOI:** 10.3389/fnbeh.2023.1080963

**Published:** 2023-02-20

**Authors:** Cristina Cadoni, Alessandra Tiziana Peana

**Affiliations:** ^1^Department of Biomedical Sciences, Institute of Neuroscience, National Research Council of Italy, Cagliari, Italy; ^2^Department of Medicine, Surgery and Pharmacy, University of Sassari, Sassari, Italy

**Keywords:** energy drinks, adolescence, alcohol, alcohol use disorders, sport, caffeine, taurine

## Abstract

Energy drinks (EDs) are beverages similar to soft drinks, characterized by high caffeine concentrations with additional ingredients like taurine and vitamins, marketed for boosting energy, reducing tiredness, increasing concentration, and for their ergogenic effect. The majority of consumers are children, adolescents, and young athletes. Although EDs companies claim about the ergogenic and remineralizing properties of their products, there is a serious lack of evidence at preclinical as well as clinical level to validate their benefits. The regular intake and long-term consequences of these caffeinated drinks are not well documented, especially the possible negative effects in adolescents whose brain is still developing. EDs combined with alcohol are also gaining popularity among adolescents and different publications indicate that this combined consumption might increase the risk to develop an alcohol use disorder, as well as produce serious adverse cardiovascular effects. There is an increasing need to disseminate knowledge on EDs damage on health, so that adolescents can be aware about the potential harmful outcomes of consuming these drinks.

## 1. Introduction

Energy drinks (EDs) are relatively new products that are like soft drinks, with additional additives and higher caffeine concentration that is the cornerstone of these beverages (Howard and Marczinski, 2010). Classic ingredients found in EDs are taurine, glucuronolactone, B vitamins, L-carnitine, sucrose, antioxidants, minerals and other herbal supplements like ginseng, guarana, yerba mate, cocoa, kola nut, and ginkgo biloba (Higgins et al., 2010). These ingredients can differ by brand as well as the list of ingredients that can be proprietary information. Although EDs companies promote EDs for energizing, anti-fatigue, concentration-boosting, and ergogenic properties, there is a serious lack of evidence and clinical trials to validate their benefits (Ruiz and Scherr, 2018).

These products are commonly consumed by adolescents and first consumption usually occurs prior to age 12 (Costa et al., 2016). Compared to girls, adolescent boys have been found to consume EDs more than once a week. Moreover, adolescents who consumed more than one ED per week were those also consuming soft drinks daily, alcohol weekly, and having high screen times or late bedtimes (Lebacq et al., 2020).

The regular intake and long-term consequences of consumption of EDs are not well recognized, most notably the possible dangerous effects on adolescents are not sufficiently studied. Moreover, research on this matter at preclinical level is still limited. Nowadays, the harmful consequences of intake of EDs are the most discussed topic of the scientific literature opening a door to awareness of possible dangers of these drinks to adolescents (Curran and Marczinski, 2017; Hladun et al., 2021; Marinoni et al., 2022).

A bibliographic search in MEDLINE/PubMed was carried out to collect clinical and pre-clinical research or review articles on EDs, and their main ingredients (caffeine, taurine, etc.), from 1987 to September 2022. A special focus was placed on EDs adverse effects (on cardiovascular system and increased risk of drug addiction) especially on children and adolescents. The reference lists of the identified articles were also scanned. Only English-language articles were reviewed. Search terms included: energy drinks/adverse effects OR energy drinks/toxicity; energy drinks/cardiovascular system; energy drinks/alcohol; energy drinks/sport; energy drinks/children OR energy drinks/adolescence; caffeine/adolescence OR caffeine/brain development; taurine/brain development; energy drinks/alcohol use disorders OR energy drinks/drug use disorders.

## 2. Energy drinks and the developing brain

Since adolescents are the main consumers of EDs (Seifert et al., 2011; Gallimberti et al., 2013) serious concerns have been raised about the detrimental effects of EDs consumption on key brain neurodevelopmental processes. Indeed, childhood and adolescence are critical periods of brain development. While white matter volume, through myelination, is increasing linearly with age, from the third trimester of gestation to the third decade of life (Giedd et al., 1999; Lebel and Deoni, 2018; Geeraert et al., 2019), gray matter volume shows an inverted “U” shaped curve, with a peak increase at adolescence (due to increased synaptogenesis) and then a decrease (due to synaptic pruning) reaching adult volume and organization of neural circuits at about 20–25 years (Lenroot and Giedd, 2006). Therefore, neuronal, and glial cells of the immature brain might be particularly vulnerable to the harmful effects of EDs consumption (Reis et al., 2017; Al-Basher et al., 2018). Caffeine and taurine appear to be the most involved in these effects (Serdar et al., 2019; Brown et al., 2020) considering the role of purinergic signaling in brain development (Rodrigues et al., 2019) and the likely role of taurine in the regulation of network excitability in the immature neocortex and hippocampus (Kilb, 2017).

Caffeine is an antagonist at A_1_ and A_2A_ adenosine receptors and thus by binding to adenosine receptors can increase the release of various neurotransmitters (glutamate, serotonin, acetylcholine, noradrenaline, dopamine) (McLellan et al., 2016) and, at high concentrations, it is also able to inhibit phosphodiesterase thereby modulating intracellular cyclic adenosine monophosphate and intracellular calcium in brain (Cappelletti et al., 2015). Evidence has accumulated in the last decades pointing to the involvement of purines in fine-tuning different processes during brain development and critical to design brain architecture (Rodrigues et al., 2019). Adenosine, through A_1_ receptor, also modulates immature synapses in different regions such as hippocampus or developing cortex (Jeong et al., 2003; Kerr et al., 2013; Burnstock and Dale, 2015). Therefore, dysfunction of purinergic signaling by caffeine overconsumption might impair brain development leading to deleterious effects, varying from mild cognitive impairment to severe neurological or psychiatric conditions (Silva et al., 2013; Al-Basher et al., 2018; Serdar et al., 2019; Zhang et al., 2020, 2022; Christensen et al., 2021; Agarwal et al., 2022).

Taurine is a non-essential free amino acid found in high concentrations in the brain, heart, and skeletal muscle (Huxtable, 1992). Taurine inhibits neuronal excitability through interaction with presynaptic glutamatergic receptors (NMDA) and GABA_A_ receptors (positive allosteric modulator of α2 subunit of GABA_A_ receptor) (Tarragon et al., 2021). In addition, taurine has high specificity and affinity for glycine receptors (Albrecht and Schousboe, 2005) and by binding to these receptors mediates glia-neuron signaling in the hypothalamus and excitatory neurotransmission in early development of the neocortex and brain stem (El Idrissi and Trenkner, 2004; Ramírez-Guerrero et al., 2022). High levels of taurine have been found in developing brain and progressively decline until adulthood (Brown et al., 2020), and evidence to date support a role in neuronal development especially in neocortical development (Kilb, 2017; Oja and Saransaari, 2022). Given that during adolescence cortical development is still ongoing and taurine levels are at the highest levels during this period, an excess of taurine intake by EDs consumption might disrupt taurine functions affecting normal development of cortical structures. Indeed, disruption of taurine homeostasis has been reported in numerous studies of neurological disorders included epilepsy and autism (Curran and Marczinski, 2017). A recent preclinical study showed the adverse effects of supplemental taurine consumption during adolescence and early adulthood on learning and memory function, suggesting an altered balance between new synapse formation and synaptic pruning during final cortical maturation as a possible mechanism (Brown et al., 2020). Indeed, taurine activation of GABA_A_ receptors may well interfere with learning and memory processes by affecting glutamate NMDA receptor pathway (Brown et al., 2020).

Another finding (Serdar et al., 2019) showed the detrimental impact of caffeine and taurine on developing oligodendrocytes and neurons. Caffeine and taurine induce degeneration and reduce proliferation of immature oligodendrocytes, accompanied by a decreased myelination capacity. Moreover, caffeine and taurine impair neuronal network formation by reducing dendrite branching and axons fragmentation demonstrating a synergistic effect of the two compounds in oligodendrocyte degeneration (Serdar et al., 2019).

## 3. Energy drinks and alcohol

**FIGURE 1:**
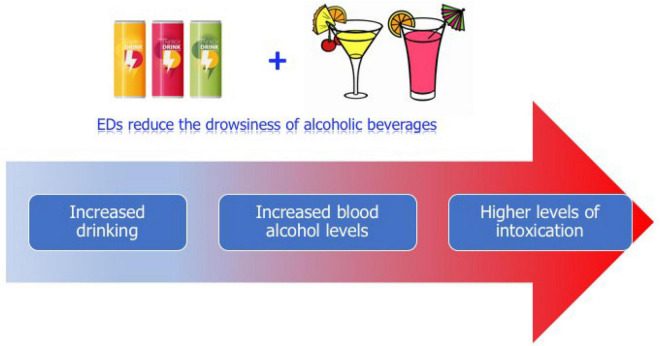
Graphical illustration of likely consequences of mixing Energy Drinks (EDs) with alcoholic drinks. The combined consumption of EDs and alcoholic beverages while reducing drowsiness induced by alcohol (perception of drunkenness) could lead to excessive alcohol intake and thus to higher blood alcohol levels causing more severe intoxications.

Some studies (Miyake and Marmorstein, 2015; Barrense-Dias et al., 2016) have identified EDs consumption at early adolescence as a predictor of using other legal and illegal substances. EDs frequently are mixed with alcoholic beverages leading to greater alcohol intake and therefore more dangerous blood alcohol levels and consequently a high-risk behavior in adolescents (Thombs et al., 2010; Figure 1). The common habit of mixing EDs with alcohol raises concern since it decreases the perception of drunkenness (Ferreira et al., 2006; Marczinski, 2014; Kaestle et al., 2018) which could lead to further fast and excessive alcohol consumption involving binge drinking and risky behaviors, as driving while intoxicated (Marczinski and Fillmore, 2014).

In a recent review analyzing the likely increased risk to develop substance use disorders by EDs consumers it has been reported that the most consistent finding is the association between EDs consumption and alcohol use disorders (AUD), while association with the use of other substances (nicotine, cannabis, stimulants, and analgesics) is controversial (Yasuma et al., 2021). Although different studies support the notion that EDs consumers are at increased risk for AUD, the possible mechanisms underlying this association is unknown (Miller, 2008; Arria et al., 2011). Some researchers postulate that EDs could function as a gateway (a priming) to drug dependence (Reissig et al., 2009). Some potential mechanisms could explain the link between these caffeinated drinks and AUD. Caffeine may prolong drinking episodes by delaying the normal sleepiness with a consequent increase in overall alcohol ingestion. On the other hand, antagonism by caffeine of the neuromodulator adenosine (Fredholm et al., 1999) might have an important role in facilitating many neuropharmacological and behavioral properties of alcohol (Sharma et al., 2010). In a recent preclinical study (Vargiu et al., 2021) it has been shown that chronic consumption of the ED Red Bull during adolescence increases dopamine in the nucleus accumbens (NAcb) shell dopamine *via* a non-adaptive mechanism, as observed with drugs of abuse. The repeated increase of shell dopamine following repeated exposure to EDs might be at the basis of a gateway effect toward abuse of other drugs.

Preclinical findings in rats, have revealed that caffeine may induce alcohol self-administration (Kunin et al., 2000; Arolfo et al., 2004). With regard to the possible mechanism of this effect, the antagonism of adenosine action by caffeine on A_2A_ receptors might be at the basis of this effect. Indeed, adenosine by activating A_2A_ receptors inhibits striatal dopamine transmission and elevation of dopamine levels is pivotal in the reinforcing properties of alcohol (as well as of all abused substances) (Nam et al., 2013; Ulenius et al., 2019).

Taurine does not affect levels of dopamine, serotonin, noradrenaline, and glutamate (Wu et al., 2017) but it can modulate dopamine neurotransmission in brain areas relevant for drug consumption (Adermark et al., 2011; Chen et al., 2018). Moreover, systemic injection of ethanol can increase taurine and dopamine in the NAcb and taurine, administered locally in the NAcb, elevates dopamine in a similar pattern as ethanol (Ericson et al., 2011). A recent study by Pulcinelli et al. (2020) shows that chronic taurine administration increases voluntary alcohol intake and preference in rats. The authors speculate that this effect might be due to an anxiolytic effect of taurine through an interaction with the GABA and dopamine systems. Relevant is the finding that acute taurine does not affect locomotor stimulation induced by alcohol administration, at variance with caffeine, but co-administration with caffeine is able to increase ethanol induced locomotor stimulation to a greater extent than each drug alone or in combination with ethanol (Ulenius et al., 2019). Since locomotor stimulation is considered an index of increased dopamine levels in the NAcb (Wise and Bozarth, 1987) and given that the increase of mesolimbic dopamine transmission is a key feature of all addictive drugs (Di Chiara et al., 2004; Di Chiara and Bassareo, 2007), it can be speculated that taurine in combination with caffeine might have a role in the increased consumption of alcohol observed in humans drinking EDs mixed with alcohol.

## 4. Energy drinks on cardiovascular system

**FIGURE 2:**
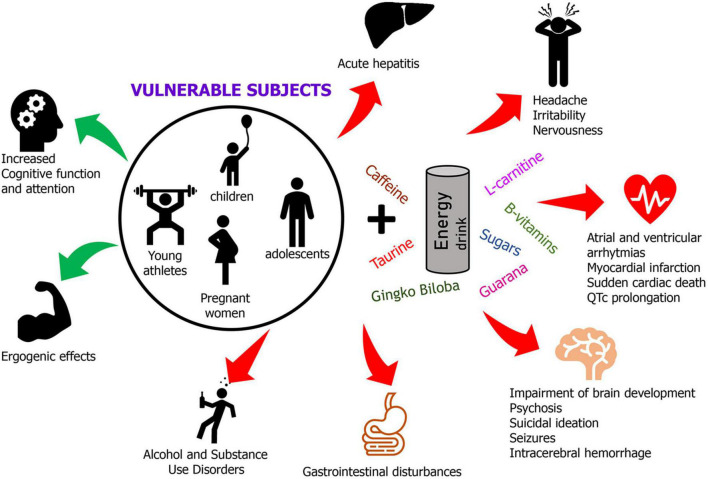
A graphical summary of positive and negative effects of EDs consumption in vulnerable individuals. In the circle are depicted the vulnerable population most sensitive to the harmful outcomes of excessive consumption of EDs. The red arrows mean negative outcomes while green arrows represent likely beneficial effects of moderate EDs consumption.

Despite claims of being safe and beneficial, consumption of EDs have been linked to fatal outcomes associated with their effects on cardiovascular system including atrial and ventricular arrhythmias, myocardial infarctions, cardiomyopathies, and sudden cardiac death (Mangi et al., 2017; Ehlers et al., 2019; Cao et al., 2021; Piccioni et al., 2021; Figure 2).

Often these negative outcomes are associated with the high caffeine concentrations in EDs, but it should be considered that other ingredients (taurine, sugars, and B-vitamins) may well contribute, through different mechanisms, to these consequences by increasing heart rate, blood pressure, and heart contractility in addition to prolonging the QTc interval (Kaur et al., 2022). When considering the potential negative outcomes of caffeine, it should be considered also the contribution of the caffeine from herbal extracts, present in EDs, such as guarana and yerba mate, whose amount is often not declared in the product label.

The mechanisms through which EDs might produce negative outcomes on cardiovascular system are different depending on the ingredient considered.

Caffeine, antagonizing adenosine action at A_2A_ receptors in vascular tissues, blocks its vasodilatory effects, increases catecholamine levels (sympathetic tone) thus increasing vascular resistance, reduces myocardial perfusion, and renin secretion. Moreover, by its competitive inhibition of phosphodiesterase, leading to an elevation of myocardial cyclic adenosine monophosphate, caffeine exerts a positive inotropic action on myocardium (Kaur et al., 2022).

As regards the possible contribution of taurine to cardiovascular negative outcomes the results of preclinical and clinical studies are inconclusive (Schaffer et al., 2014; Ehlers et al., 2019). This might be due to different effects of taurine alone or in combination with caffeine and depending on dose and acute or long-term exposure. Although several beneficial effects have been attributed to taurine in different studies as anti-inflammatory, antioxidant, and neuroprotective in neurodegenerative disorders, antiarrhythmic, and hypotensive activity (Schaffer et al., 2014; Caine and Geracioti, 2016), when taurine is ingested with caffeine led to an increase in blood pressure (Grasser et al., 2016). A recent study has investigated a likely role of taurine combined with caffeine in inducing arrhythmias in an animal whole-organ model. The authors found that low doses of taurine decreased the number of ventricular arrhythmias whereas higher concentrations provoked significantly more arrhythmias in a dose-dependent fashion (Ellermann et al., 2022). This finding is not surprising based on the inotropic effect of taurine on myocardium (Schaffer et al., 2014) consistent with the ability of taurine to release intracellular calcium thus having a role in the regulation of calcium homeostasis and contractile function (Ehlers et al., 2019).

Most of EDs consumed by adolescents contain high amounts of sugars with a variable combination of glucose, sucrose, fructose, or high corn-fructose syrup. Among these, fructose has the greatest autonomic effect, increasing blood pressure after consumption (Somers and Svatikova, 2020). Glucose, fructose, and sucrose also increase heart rate after ingestion and caffeine may have a synergistic effect by increasing blood glucose and insulin after consumption (Nowak et al., 2018). These effects might exacerbate not only cardiovascular effects of caffeine but also, with excessive and long-term use, lead to obesity, insulin resistance, and diabetes, with the latter being a risk factor for cardiovascular diseases.

B-vitamins (thiamine, riboflavin, niacin, pantothenic acid, pyridoxine hydrochloride, biotin, inositol, and cyanocobalamin) are involved in many metabolic processes essential for a proper cell function, especially mitochondrial function and energy production (Depeint et al., 2006; Kennedy, 2016). Since EDs contain large amounts of sugars, addition of these vitamins has likely the purpose to provide cofactors for energy utilization and metabolism. However, despite the beneficial effects of these vitamins, it cannot be ruled out any adverse effect of an overload of these nutrients (Seifert et al., 2011; Wolk et al., 2012; De Sanctis et al., 2017). Indeed, while it was reported a beneficial effect of vitamin B_12_ (cyanocobalamin) in reducing homocysteine levels and thus cardiovascular risk, recently it has been suggested that an excess of vitamin B_12_ might have harmful cerebrovascular consequences (Duschek et al., 2016). Among the effects of EDs consumption on cardiovascular system it should be included the increased platelet aggregation and endothelial dysfunction leading to risk of thrombosis (Worthley et al., 2010). This effect could not be related to caffeine (Natella et al., 2008) while many different ingredients of EDs may well interact to produce this negative outcome on platelet and endothelial function (Olas and Bryś, 2019).

## 5. Sport and energy drinks

The ergogenic effect of EDs is mainly attributed to caffeine content, although all the other ingredients might potentiate this effect. Until 2004, caffeine was in the doping list of substances banned by the World Anti-Doping Agency. Since its removal from the banned substances list caffeine has been used by athletes in any quantity and form without the burden of being sanctioned. Evidence supports the ergogenic effects of these caffeinated drinks, involving (1) prolonged endurance exercise; (2) muscle strength and muscle endurance; and (3) high-intensity exercise and intermittent sprints (McLellan et al., 2016). The ergogenic effect seems similar in male and female athletes. However, their efficacy is linked to the need of a minimum intake of caffeine of 3 mg/kg (Jiménez et al., 2021), considered the optimal dose for the ergogenic effects (Guest et al., 2021). Nonetheless, often this dose is overcome thus raising concerns about the risk-benefit ratio given the side effects on cardiovascular function (de Souza et al., 2022).

The efficacy of caffeine and EDs on sports performance mainly depends on various factors such as timing and dose consumed, the type of sport, and the individual response to caffeine (Burke, 2008). Caffeine seems to be effective in long-lasting sport activities, with the greatest effects in sports involving fatigue during or toward the end of the event, while it does not seem to be effective in single events short lasting, involving strength and power, requiring very high intensity (Burke, 2008).

Other components of EDs may act synergistically with caffeine in increasing physical performance. Taurine modulates skeletal muscle contractile function and may attenuate exercise-induced DNA damage, with some evidence showing the ability to improve exercise capacity and performance (Higgins et al., 2010). Moreover, since taurine plays a role as an antioxidant it could improve ATP turnover in the muscle cell, leading to an increase in high intensity exercise performance (Karayigit et al., 2021). L-carnitine, another component of EDs, plays an important role in reducing inflammatory responses, oxidative stress, and apoptosis. Thus, preventing cellular damage L-carnitine favorable affects recovery from exercise stress. It has been shown that dietary supplementation with L-carnitine increases maximal oxygen consumption and lowers the respiratory quotient, indicating stimulation of lipid metabolism (Karlic and Lohninger, 2004).

## 6. Side effects of EDs in adolescents

In addition to the dangerous consequences on cardiovascular system, other systems have been reported to be negatively affected by EDs consumption. Among acute side effects observed following EDs consumption there are headache, irritability, excitability, malaise, dehydration, nervousness, insomnia, nausea/vomiting, abdominal pain (Wolk et al., 2012; Nadeem et al., 2021; Khouja et al., 2022). More serious and even fatal consequences have been reported following large amount of EDs including seizures, intracerebral hemorrhage, acute hepatitis, acute renal failure, psychosis, suicidal ideation (Hernandez-Huerta et al., 2017; Kim et al., 2018; Kelsey et al., 2019), Figure 2. The side effects associated with EDs consumption are mainly related to their caffeine content (Breda et al., 2014). In adolescents, who are not usual caffeine consumers, susceptibility to caffeine intoxication could be significantly augmented due to a lack of pharmacological tolerance (Thombs et al., 2010; Thomson et al., 2014). In addition, genetic factors could similarly contribute to an individual susceptibility to caffeine illnesses as well as caffeine intoxication, dependence, and withdrawal (Turagam et al., 2015). Compounds such as guarana, yerba mate, cocoa, and kola nut may increase biological effects of caffeine in EDs (Babu et al., 2008). In particular, guarana and caffeine, alone or in association with taurine, could induce neurotoxicological effects as well as an interference on redox homeostasis (Zeidán-Chuliá et al., 2013). In a case report, the consumption of EDs in adolescents, produced a dental enamel erosion resulting from the acidity of EDs (Duchan et al., 2010).

## 7. Conclusions

The use of EDs is an issue hotly debated due to concerns regarding the consumption by adolescents of high caffeine amounts with consequent deleterious effects on the developing brain and cardiovascular systems. Avoidance of caffeine use in young people poses a great societal challenge because of the widespread availability of caffeine-containing beverages and a lack of awareness of the potential risks.

The present review focused mostly on insidious nature of EDs consumption given the likely dangerous effects on adolescents and deleterious outcomes of their ingredients on developing brain. Although clinical research points to the negative effects of EDs in adolescents, there is a need for further studies. Among current research gaps there is the fact that most of clinical studies are indeed cross-sectional and therefore not suitable to draw definitive conclusions since the cause or effect cannot be determined where an association is found. In this regard longitudinal studies could provide stronger evidence. Moreover, although pre-clinical research is helping to elucidate the negative outcomes of EDs in adolescents, it is still scarce and not exhaustive. Notably, different studies are conducted on EDs *in toto* and not on the singular components at well-defined concentrations and this is a significant critical issue precluding identification of any synergism between components.

Future development in the field should take into account the above weaknesses providing more information on acute and long-term effects of the use of EDs. There is a need of spreading the knowledge on EDs damage on health to let adolescents be aware that these drinks are potentially devious.

## Author contributions

CC and AP equally contributed to the literature review and in writing the manuscript. Both authors were responsible for manuscript revision and approved the submitted version.
